# High prevalence of HIV infection and unprotected anal intercourse among older men who have sex with men in China: a systematic review and meta-analysis

**DOI:** 10.1186/1471-2334-14-531

**Published:** 2014-10-06

**Authors:** Yong-ze Li, Jun-jie Xu, Han-zhu Qian, Bing-xin You, Jing Zhang, Jian-ming Zhang, Qing-hai Hu, Zhen-xing Chu, Shu-yang Liu, Yong-jun Jiang, Wen-qing Geng, Hong Shang

**Affiliations:** Key Laboratory of AIDS Immunology of National Health and Family Planning Commission, Department of Laboratory Medicine, The First Affiliated Hospital, China Medical University, Shenyang, Liaoning, China; Collaborative Innovation Center for Diagnosis and Treatment of Infectious Diseases, Hangzhou, China; Vanderbilt Institute for Global Health, Vanderbilt University, Nashville, TN USA; Division of Epidemiology, Department of Medicine, Vanderbilt University Medical Center, Nashville, TN USA; Department of Finance, Business College, Washington State University, Pullman, WA USA; Department of Clinical Epidemiology and Evidence-based Medicine, The First Affiliated Hospital, China Medical University, Shenyang, Liaoning, China

**Keywords:** HIV, Syphilis, Unprotected anal intercourse (UAI), Men who have sex with men (MSM), Older MSM, China

## Abstract

**Background:**

China has the largest number of the elderly in the world. As the proportion of elderly is rapidly increasing among national reported HIV/AIDS cases, it is a concern about HIV epidemic among older MSM in China. However, studies on HIV prevalence and unprotected anal intercourse (UAI) among Chinese older MSM were relatively few or generally had small sample sizes.

**Methods:**

English and Chinese articles published in peer-reviewed journals were identified by systematically searching 5 electronic databases including PubMed and through cross-referencing. Summary prevalence rates of HIV infection and UAI with male sexual partners were calculated, and analyses were performed using the software Comprehensive Meta-Analysis V2.0 and SPSS V17.0. Subgroup analyses were performed separately by sample size, study year, study location, recruitment settings and sampling method.

**Results:**

Twenty eligible cross-sectional studies (3 in English and 17 in Chinese), published between 2005 and 2013, with a total of 2812 older MSM participants, were identified. Our meta-analyses showed that the prevalence of HIV, syphilis and UAI in the last 6 months were 11.6% (95% confidence interval [CI]: 8.0%-16.6%), 23.0% (95% CI: 15.8%-32.3%) and 79.5% (95% CI: 72.7%-84.9%), respectively. HIV prevalence increased over the study period (6.3% in 2003–2007; 8.6% in 2008–2009, and 11.5% in 2010–2011, trend test Chi-square = 7.02, p = 0.008). The pooled prevalence of HIV (11.6% vs. 5.2%, Chi-square value = 36.2, p < 0.001) and UAI (79.5% vs. 52.6%, Chi-square value = 440.04, p < 0.001) among older MSM were both significantly higher than among younger age group (age < 50 years).

**Conclusions:**

Older Chinese MSM have high prevalence of HIV and syphilis. Unprotected anal sex is common and further puts them at high risks of acquiring and transmitting HIV, which was one of reasons for the rapid increasing of national reported older male HIV/AIDS cases. Prevention intervention programs should be specially tailored for this high risk MSM subgroup.

**Electronic supplementary material:**

The online version of this article (doi:10.1186/1471-2334-14-531) contains supplementary material, which is available to authorized users.

## Background

Human immunodeficiency virus (HIV) infection and other sexually transmitted infections (STIs) continue to disproportionately affect men who have sex with men (MSM) in China [1]. A study conducted in 61 cities in China between February 2008 to September 2009 found that 4.9% of MSM were infected with HIV [2]. According to a report from the Chinese Ministry of Health, it estimated that there were approximately 780,000 people living with HIV/ AIDS in 2011 in China [3], 17.4% of the estimated HIV/AIDS cases were attributable to male-to-male sexual contact. Sentinel surveillance data have shown an increasing trend of HIV infection among MSM, from 2.0% in 2007 to 6.3% in 2011 [4].

China has the largest number of the elderly in the world [5]. According to a report from the China National Committee on Ageing, there were an estimated 185 million elderly people (aged 60 years or older) in China by the end of 2011, and about half are men [6]. Chinese elderly people were generally not considered as a population at high-risk for HIV infection [7]. Whereas, among nationally reported HIV/AIDS cases, the proportion of total cases accounted for by the 50–64 age group increased from 1.6% to 13.8% in 2000 and 2011, respectively [4].

As the number of new HIV cases amongst the 50+ age group increased noticeably in recent years, there have been a few studies exploring the reasons in recent years, and shown that engaging in commercial sexual activities after their retirement is a major factor for the increased HIV epidemic among the older men [8, 9]. As MSM has become a major at-risk group, it is a concern about HIV among older MSM in China. However, studies on HIV prevalence and unprotected anal intercourse (UAI) among Chinese older MSM are relatively few and generally have small sample sizes [7, 10, 11]; it will be helpful for assessing the magnitude of HIV epidemic and risk behaviors in this age group through summarizing the findings from these studies. We performed Meta analysis of the prevalence of HIV, syphilis and unprotected anal sex (UAI) in the last 6 months among older MSM in China.

## Methods

### Search strategy

Studies published in English or Chinese were identified through searching the following electronic databases: PubMed, China National Knowledge Infrastructure (CNKI), Chinese Scientific Journals Full text Database (CQVIP), Wanfang and Google Scholar. The keywords and medical subject headings “HIV”, “AIDS”, “MSM”, “gay”, “homosexual”, “elderly”, “older”, “aged”, “old”, and “China” were used to search for potentially relevant studies (e.g. http://www.ncbi.nlm.nih.gov/pubmed/?term=old+MSM+HIV+China). The last date of search was July 1st 2013. This meta-analysis was conducted in accordance with the guidelines of the Preferred Reporting Items for Systematic Reviews and Meta-Analyses statement (PRISMA) [12].

### Inclusion and exclusion criteria

Studies were eligible for inclusion in the review if they met the following criteria: (1) published in Chinese or English language; (2) conducted in China; (3) study participants aged ≥ 50 years old MSM; (4) reported homosexual behaviors (recall window ≤ 6 months); (5) presented laboratory diagnosis of HIV and/or syphilis, as well UAI which was defined as no condom use in insertive or receptive anal sex for at least once in the past 6 months.

Studies were excluded if they met the following criteria: (1) not an original study, e.g., review or editorial; (2) not a peer review journal article, e.g., government report or conference abstract; (3) if the same study data were published in both English and Chinese, Chinese publications were excluded from the review, and (4) studies that exclusively recruited MSM participants with specific behaviors that may inflate the estimation of the prevalence of HIV or syphilis or UAI, e.g., money boys, illegal drug users.

### Data extraction and study quality assessment

For all eligible studies, two reviewers (YL and BY) independently extracted the following data from original publications: first author and year of publication; study location and study year; sampling method and recruitment setting; sample size; and results of HIV and syphilis tests and UAI prevalence (if available). Disagreements between the two reviewers during data extraction were reconciled by a third reviewer (JX).

The quality assessment checklist for observational studies (QATSO score) was selected to evaluate the quality of the included studies [13]. Items were scored as 1, 0, NA, which corresponded to “yes”, “no”, or “not applicable”, respectively. The total score was divided by the total number of all applicable items. Values between 0%- 33%, 34%-66%, and 67%- 100% represent “bad”, “satisfactory” and “good” quality, respectively [13].

### Statistical analysis

Standard meta-analytic approaches were used in calculating prevalence estimates for individual studies and for aggregating estimates across studies [14]. Meta-analysis of the prevalence of HIV and syphilis infections and UAI was carried out using the Comprehensive Meta-Analysis software (V2.0, Biostat, Englewood, NJ). The pooled prevalence was calculated using the inverse variance method. The average effect size across all studies is computed as a weighted mean, whereby the weights are equal to the inverse variance of each study’s effect estimator [15]. Larger studies and studies with less random variation are given greater weight than smaller studies [15].

We also tested the heterogeneity of the findings across studies by using the Q statistic. The aggregated results were calculated based on a random-effects model, which provides a more conservative estimate of variance and generates more accurate inferences about a population of studies beyond those included in this review. The Begg rank correlation method was used to assess the potential for publication bias (p < 0.05 was considered the indicative of statistically significant publication bias). Analyses were conducted separately for HIV, syphilis and UAI.

Stratified analyses were conducted to examine whether the prevalence of HIV infection differed by study location, study year, sample size, recruitment setting and sampling method. The changes of HIV prevalence over the study period and the difference between the pooled prevalence of HIV and UAI among older MSM and their younger counterparts were analyzed for linear trend using SPSS (V17.0, Chicago, IL, USA). Their younger counterparts were defined as the total MSM minus the older MSM within the studies.

In the sensitivity analyses, we compared the overall estimate with the estimates obtained after iterations using k-1 studies (k = number of independent samples). We removed a study and calculated the overall prevalence estimate. Then, we replaced that study, removed another, and repeated the process.

## Results

### Study selection

**Figure 1:**
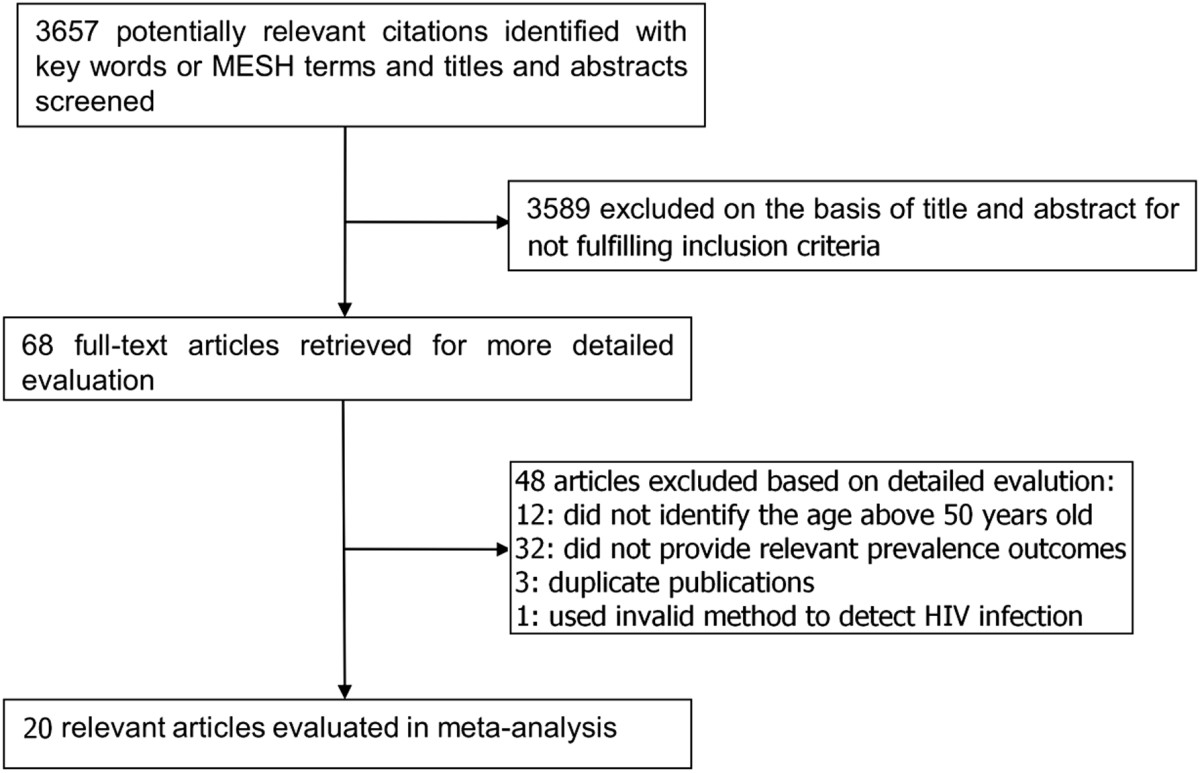
**Flow diagram of the study selection process.** As shown, our initial searches yielded 3657 citations from Pubmed, Chinese National Knowledge Infrastructure, Wanfang and Google Scholar databases. After screening titles and abstracts, 68 studies were considered potentially eligible and retrieved in full text. Of these, 48 studies were subsequently excluded because they did not satisfy the inclusion criteria. Thus, 20 fully eligible studies were identified.

A total of 3657 citations were identified, of which 68 potentially relevant articles were selected for further screening, and eventually 20 articles (3 published in English and 17 in Chinese) with 2812 older MSM participants met the inclusion criteria (Figure 1). Besides, we identified 59556 younger MSM (age < 50 years) as their comparison. Table 1 provides a descriptive summary of these studies [2, 16–34]. All studies had laboratory testing results of HIV infection, 7 had results of syphilis infection, and 11 measured UAI. All the included studies met the criteria of “good quality” (values between 67% and 100%) (Additional file 1).

**Table 1:** **Prevalence of HIV infection among older MSM in China**

First author, published year	Study design				Older MSM				Younger MSM			
	Location	Study year	Sampling method	Recruitment settings	Sample size	HIV infection^a^	Syphilis infection^b^	UAI	Sample size	HIV infection	Syphilis infection	UAI
Ma AB, 2007	Yunnan	2005	Mixed^c^	Medical settings	8	1	NR^d^	NR	70	7	NR	NR
Xi SJ, 2011	Hangzhou	2009-2010	Snowballing	Multiple gay venues or events	16	1	NR	NR	514	44	NR	NR
Long QP, 2012	Hunan	2011	Mixed	Multiple gay venues or events	33	7	NR	27	233	36	NR	178
Zhou JB, 2012	Changzhou	2007	Snowballing	Multiple gay venues or events	43	12	18	NR	550	69	167	NR
Wang ZC, 2012	Xining	2011	Mixed	Medical settings	21	2	0	17	382	45	40	233
Qun He, 2006	Guangzhou	2003	Mixed	Medical settings	23	0	NR	NR	201	0	NR	NR
Xuan ZB, 2012	Shanghai	2010-2011	Snowballing	Medical settings	9	0	NR	7	89	3	NR	64
Chu ZX, 2011	Shenyang	2008	Snowballing	Medical settings	56	4	11	50	2018	96	234	1888
Yan Xiao, 2010	20 cities	2006	Snowballing	Multiple gay venues or events	1009	55	NR	834	2961	91	NR	2034
Wu ZY, 2013	61 Cities	2008- 2009	RDS^e^	Medical settings	1182	90	274	813	45478	2196	5226	23294
Zhou YQ, 2012	Shanghai	2010- 2011	Mixed	Medical settings	6	1	2	NR	288	17	36	NR
Yang LG, 2012	Fuyang	2010-2011	Mixed	Multiple gay venues or events	2	0	NR	2	263	15	NR	163
Lan GH, 2009	Guangxi	2008	Snowballing	Medical settings	41	2	5	28	1105	18	60	613
Feng F, 2009	Haikou	2008	Mixed	Medical settings	4	0	NR	NR	100	2	NR	NR
Zhang FX, 2011	Suzhou	2008- 2009	Mixed	Medical settings	38	2	NR	27	616	56	NR	384
Li R, 2010	Dalian	2009	Snowballing	Multiple gay venues or events	6	2	NR	NR	396	16	NR	NR
Chen Y, 2013	Guizhou	2008- 2009	Mixed	Multiple gay venues or events	32	14	NR	27	798	179	NR	513
Ni ZM, 2011	Hangzhou	2009	Mixed	Multiple gay venues or events	5	1	1	NR	214	33	43	NR
Xu J, 2010	4 cities	2008	RDS	Medical settings	252	24	NR	214	1612	101	NR	1259
Zheng LX, 2012	Longyan	2010- 2011	Snowballing	Multiple gay venues or events	26	2	NR	NR	377	13	NR	NR

^a^Blood specimens were detected by Enzyme-Linked Immuno Sorbent Assays (ELISA) and Western Blot assay (WB) to confirm HIV infection in all the included studies; ^b^Blood specimens were detected by Rapid Plasma Reagin (RPR) and Treponema Pallidum Particle Agglutination assay (TPPA)to confirm syphilis infection in all the included studies; ^c^Mixed: Snowballing or/and RDS or/and convenience sampling were included; ^d^NR: Not reported; ^e^RDS: Respond Driven Sampling.

### Prevalence of HIV and syphilis infection

**Figure 2:**
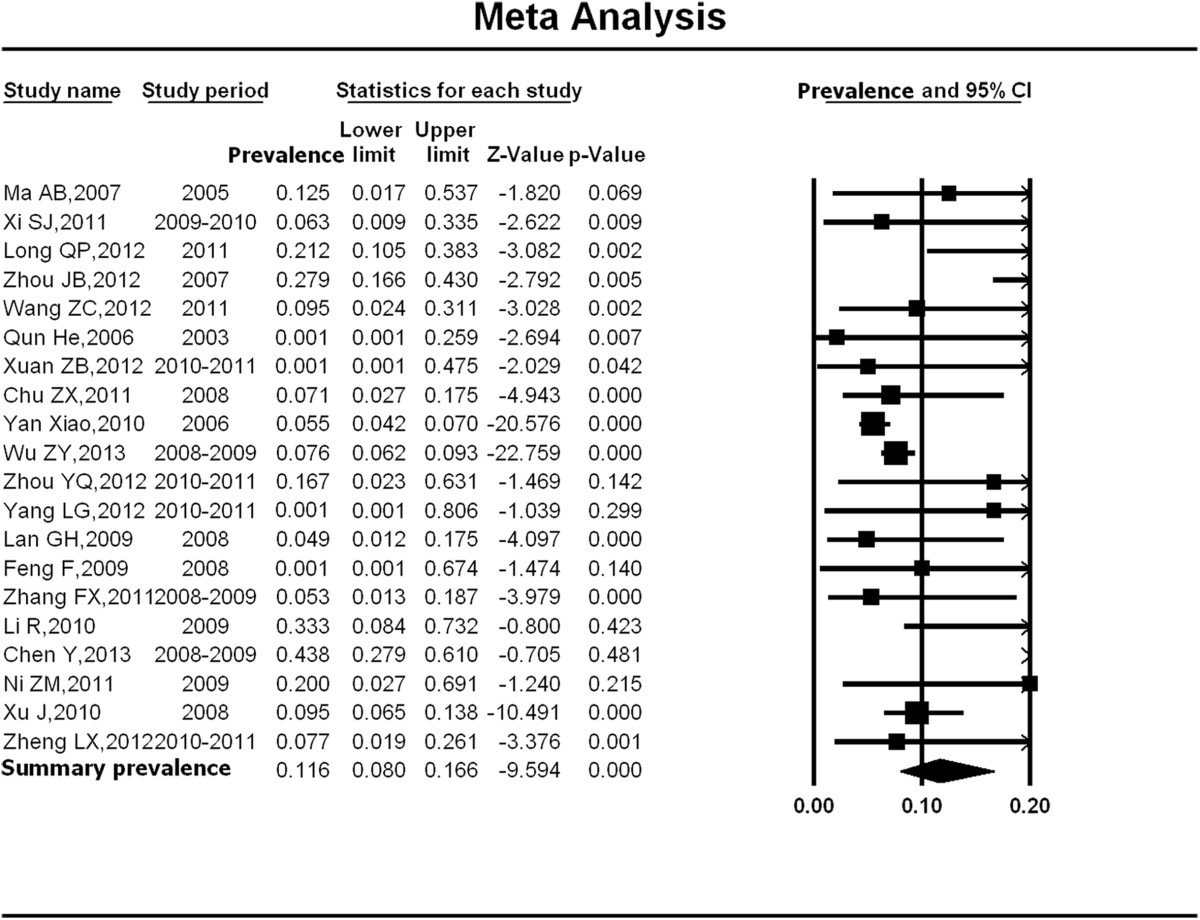
**Meta-analysis of the HIV prevalence among older MSM in China.** Figure 2 shows unadjusted HIV prevalence estimates (boxes) with 95% confidence limits (bars) for each study selected; pooled prevalence estimates are represented as diamonds in this plot.

Figure 2 shows the summarized estimates of HIV prevalence among older MSM from 2003–2011 (11.6%; 95% confidence interval [CI]: 8.0%-16.6%) with the prevalence rate in individual studies ranging from 2.1% (95% CI: 0.1%-25.9%) to 43.8% (95% CI: 27.9%-61.0%). The summary HIV prevalence among older MSM was significantly higher than their younger counterparts (11.9% vs. 5.1%, Chi-square value = 40.3, p < 0.001). No significant publication bias was observed (p = 0.77). However, there was substantial heterogeneity between studies (p for Q test, p < 0.01; *I*^2^ = 76.51).

**Figure 3:**
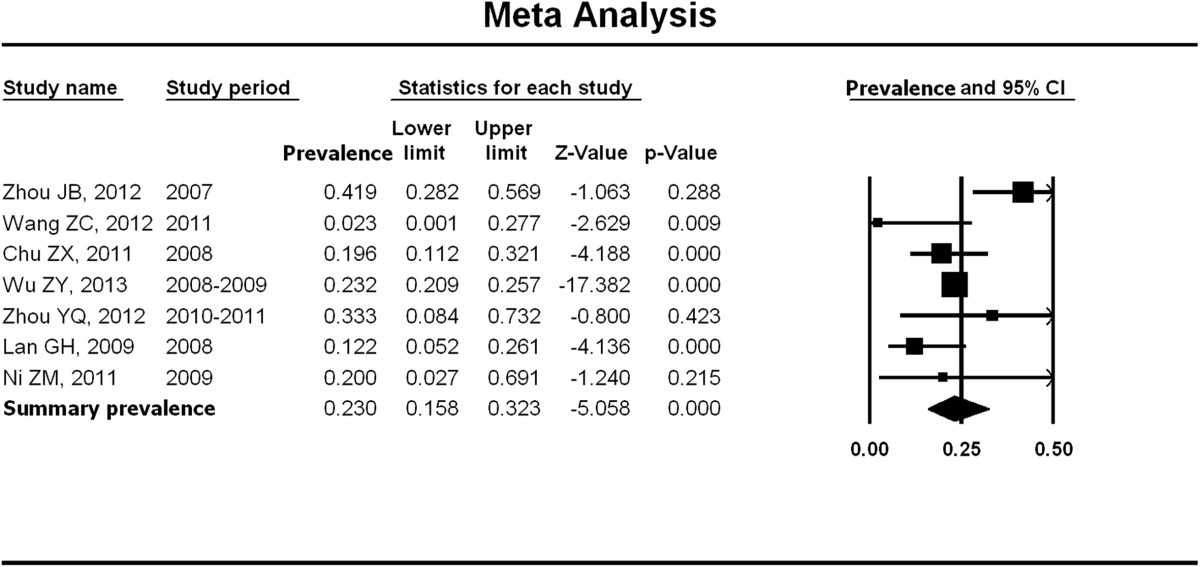
**Meta-analysis of syphilis prevalence among older MSM in China.** Figure 3 shows unadjusted syphilis prevalence estimates (boxes) with 95% confidence limits (bars) for each study selected; pooled prevalence estimates are represented as diamonds in this plot.

Figure 3 shows the summarized prevalence of syphilis infection, of the average prevalence was 23.0% (95% CI: 15.8%-32.3%) with a range from 2.3% (95% CI: 0.1%-7.7%) to 41.9% (95% CI: 28.2%-56.9%). The summary prevalence of syphilis infection among older MSM was marginal significantly higher than their younger counterparts (23.0% vs. 10.0%, Chi-square value = 3.57, p = 0.06).There was medium heterogeneity between studies (p = 0.03; *I*^2^ = 58.59). No publication bias was observed as assessed by the Begg rank correlation analysis (p = 0.37).

### Stratified analyses and trend test of HIV prevalence

**Figure 4:**
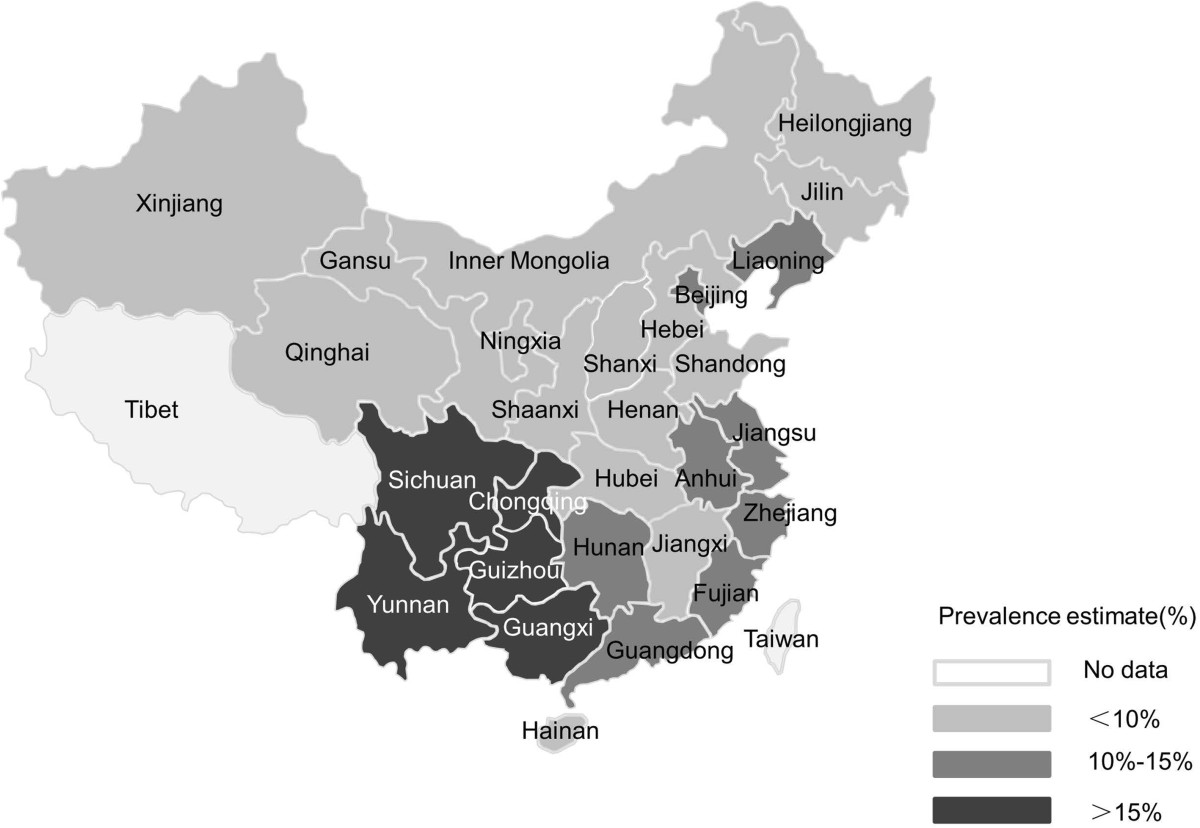
**The regional distribution of pooled prevalence of HIV among older MSM in China.** Created by: YZL. Generated by: Microsoft Office PowerPoint.

Table 2 presents the findings of stratified analyses, which were conducted for study location, study year, sample size, recruitment setting and sampling method. Heterogeneity between studies was partly reduced in subgroup analyses. The subgroup of studies with larger sample size (n > 200) had a lower prevalence than smaller studies (7.2% vs. 13.6%). More recent studies found a higher prevalence than older studies: 11.5% (13/113) in 2010–2011, 8.6% (139/1616) in 2008–2009 and 6.3% (68/1083) in 2003–2007. The trends over these three time periods are statistically significant (trend test Chi-square value = 7.02, p = 0.008). The highest HIV prevalence was reported in the studies conducted in southwest China (including the provinces of Yunnan, Guangxi, Sichuan, Guizhou, and the city of Chongqing), and the lowest prevalence was reported from nationwide studies (also see Figure 4). The summary prevalence in the southwest was 16.4%, followed by north China (14.7%), east China (13.0%), south China (12.3%), west China (9.5%), and nationwide (7.2%). The summary HIV prevalence was significantly higher in studies that recruited participants from gay venues or events than from medical settings (17.4%, vs. 7.9%), and was higher in studies using mixed sampling methods (15.5%) than using respondent driving sampling (RDS) (8.0%) and snowball sampling (9.8%).

**Table 2:** **Stratified meta-analyses of HIV prevalence among older MSM of China**

Subgroups	Prevalence % (95% CI)	No of studies	Heterogeneity	
			I^2^%	p Value
Sample size				
> 200	7.2%(5.4%-9.5%)	3	70.67	<0.05
≤ 200	13.6%(8.4%-21.2%)	17	55.86	<0.05
Study year				
2003-2007	9.9%(2.7%-30.6%)	4	89.30	<0.05
2008-2009	12.1%(6.8%-20.5%)	9	81.04	<0.05
2010-2011	13.6%(8.2%-21.8%)	7	—	>0.05
Study location				
East China	13.0%(6.8%-23.4%)	8	32.12	>0.05
Nationwide	7.2%(5.4%-9.5%)	3	70.67	<0.05
North China	14.7%(2.7%-51.5%)	2	70.91	>0.05
South China	12.3%(3.3%-36.6%)	3	35.25	>0.05
Southwest	16.4%(2.8%-57.4%)	3	83.90	<0.05
West China	9.5%(2.4%-31.1%)	1	—	<0.05
Recruitment settings				
Medical settings	7.9%(6.7%-9.3%)	11	—	>0.05
Multiple gay venues or events	17.4%(7.6%-35.0%)	9	89.20	<0.05
Sampling method				
Mixed	15.5%(8.0%-27.8%)	10	52.88	<0.05
RDS	8.0%(6.7%-9.6%)	2	2.98	>0.05
Snowballing	9.8%(4.7%-19.2%)	8	78.12	<0.05

### Prevalence of unprotected anal intercourse with male partners

**Figure 5:**
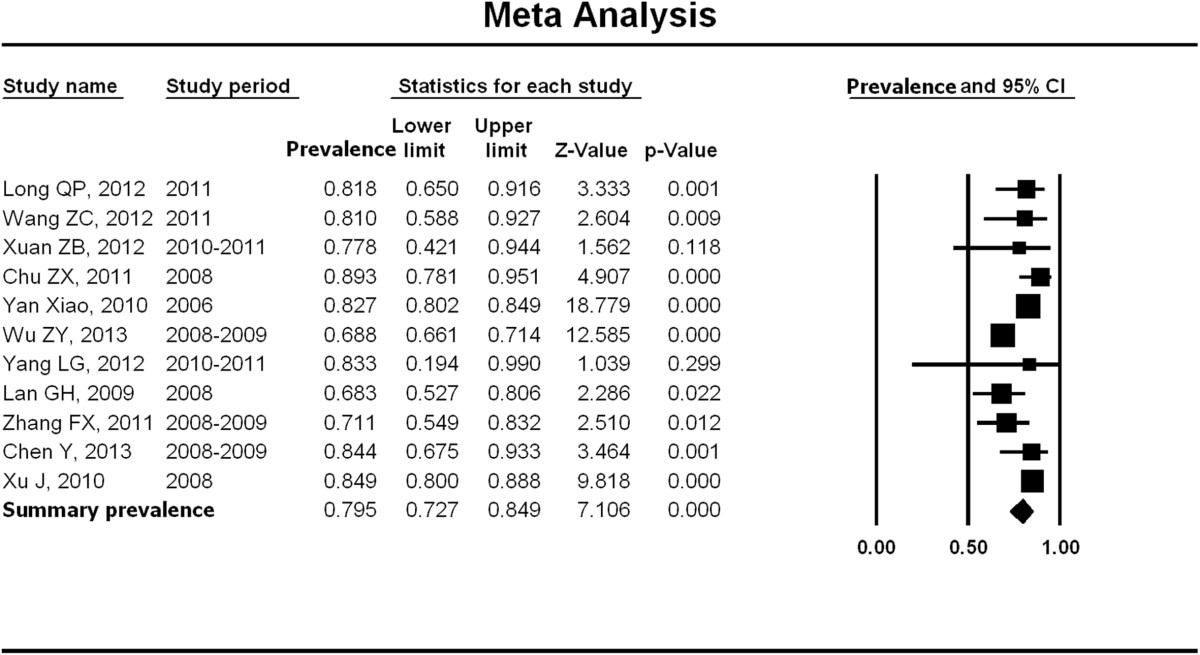
**Meta-analysis of UAI prevalence among older MSM in China.** Figure 5 shows unadjusted UAI prevalence estimates (boxes) with 95% confidence limits (bars) for each study selected; pooled prevalence estimates are represented as diamonds in this plot.

The aggregated findings from 21 studies showed that the prevalence of having ever engaged in UAI with any male partner in the past 6 months was 79.5% (95% CI: 72.7%-84.9%) (Figure 5). There was significant heterogeneity of results across studies (p < 0.01; *I*^2^ = 87.03). However, sensitivity tests did not reveal any individual study that exerted influence on the overall estimate. There was no evidence of publication bias (p = 0.76). The difference between the pooled prevalence of UAI with older MSM was significantly higher than their younger counterparts (79.8% vs. 61.7%, Chi-square value = 238.2, p < 0.001).

## Discussion

To our knowledge, this meta-analysis provides the first quantitatively synthesized estimates of the prevalence rates of HIV, syphilis and UAI among older MSM in China. The summarized estimates were HIV (11.6%; 95% CI: 8.0%-16.6%), syphilis (23.0%; 95% CI: 15.8%-32.3%) and UAI (79.5%; 95% CI: 72.7%-84.9%). Our meta-analysis also demonstrated that the trend of HIV prevalence among older MSM has substantially increased in the past decade. Besides, our analysis also provided evidence contradictory to a widely accepted assumption that young MSM was at higher HIV risk than older MSM [35, 36]. The overall and stratified estimates provide useful formation for epidemiologic modeling of HIV transmission among older MSM and for allocating resource for targeted prevention interventions.

HIV prevalence was higher among older MSM than their younger counterparts (11.6% vs. 5.2%), and is higher than average prevalence (4.9%) among nearly 50,000 MSM in a nationwide survey during 2008 and 2009 [2], and is also higher than the average prevalence among national sentinel sample (6.3% for HIV, and 10% for syphilis) [3]. Firstly, it is possible that older MSM had longer durations of exposure to HIV source. Secondly, HIV testing as increased HIV prevalence may be to some extent due to increase testing rate since HIV testing among Chinese MSM has been increasing over the decade [37], then antiretroviral treatment could also contribute to a higher prevalence among older MSM as they just age with their infection. In addition, UAI among older MSM was also more common than their younger counterparts, which might partly explain the higher HIV prevalence among older MSM. HIV prevalence among older MSM in China is higher than older MSM in European countries such as Sweden (3.8%) and Germany (2.6%), but was much lower than those in four major cities in the United States (19%) [38, 39].

Syphilis infection is a good surrogate for high risk sexual behaviors. A high prevalence of syphilis (23.0%) among Chinese older MSM suggested that these men may have heavily engaged in unprotected sex, which could facilitate HIV spread in this group, and screening and treating syphilis could be an effective way prompting HIV detection [40]. Syphilis itself would also act as a cofactor for HIV acquisition [41–44]. Due to the mode of sexual transmission, single public health intervention campaign can be designed with the objectives of preventing HIV and preventing syphilis. Although UAI is common among older MSM, HIV has not yet reached the high level as syphilis. We interpret this as a significant opportunity to save lives through increasing public health intervention efforts [2].

We also found that a sizeable percentage (79.5%) of older MSM had engaged in UAI with male partners. This is an important public health concern, given the high prevalence of HIV and STIs among older MSM in China. The UAI prevalence was higher among older MSM than among their younger counterparts, which was generally consistent with several recent studies in China [11, 45], but it is contradictory to the studies in the United States [46].

Subgroup analysis showed the highest HIV infection rate in southwest China (16.4%), which was consistent with prior studies [47, 48].

Older MSM living with HIV may bring more medical challenges. The elders are more vulnerable to side effects of antiretroviral drugs [49]. Aged people are facing problems of diabetes and heart disease, whereas research suggests that antiretroviral drugs contribute to high cholesterol levels and hamper insulin production, thus increasing the risk of health problems [50]. Besides, significant HIV epidemic among older MSM is not only a medical issue but also a social problem. Due to traditional Chinese values, the majority was married, but they continued engaging in extramarital sex with their male partners. Studies have shown that they are less likely to take precautions [51], even though they know HIV risk from unprotected sex [52], because they have misconception that HIV/AIDS is a disease of young people [53]. What is worse, some of them hold opinion perception that the latent time from infection to development of AIDS could be up to ten years, and they may die of other diseases, so they do not even care about HIV/AIDS [54]. Little HIV prevention work has focused on older MSM, partly due to the widespread belief that older MSM are not sexually active [46]. Our analysis provides strong evidence of the need for interventions in this subgroup. Formative work is needed to determine appropriate approaches for this population. Some of the older MSM are likely to engage in bisexual behaviors, which may play a bridging role in the spread of HIV and other sexual transmitted diseases from this high-risk group to the general population [55]. Moreover, the alarming spread of HIV among older MSM is fuelled by an ongoing, persistent stigma against homosexuality in China [56]. This may lead to a result that older MSM hide their sexual identity, thereby increasing the difficulty of reaching them by prevention interventions. It is critical to reduce stigma against homosexuality and mitigating discrimination against homosexuals among the general population.

Our meta-analytic findings should be viewed within the context of the methodological limitations of the primary studies. Our findings are based on cross-sectional data, which provide snap shots of prevalence of HIV, syphilis, and UAI at the time of data collection. The included studies are heterogeneous in term of sample size; recruitment setting, sampling method, study year, and study location. For example, some studies recruited participants through RDS or snowball sampling rather than random sampling, and these sampling approaches may have selection bias [40]; More studies were conducted in east China, and few in west China. Therefore, the summary estimated may not represent the national sample. Small sample size may also restrict the statistical power in subgroup analyses, there are very small sample sizes of many studies and probably also bias in some studies. Though no major publication bias was indicated in the meta-analysis, the possibility of publication bias could not be fully excluded. In the stratified analyses, heterogeneity between studies was reduced, suggesting that the varied data collection method, recruitment setting, sample size, study time and study locations may account for the inconsistency among studies. A higher prevalence of HIV infection was found among studies with sample sizes ≤ 200 compared with sample sizes >200, and this may suggest the prevalence may be overestimated in the studies with a smaller sample size.

## Conclusion

Our meta analysis provided important findings on HIV and syphilis epidemics and UAI among older MSM in China. These findings are contradictory to the widespread perception that young MSM are at the highest risk of HIV infection. Older MSM have been ignored in HIV/STI prevention intervention programs, and more focus should be given to this neglected and hard-to-reach subgroup in future HIV interventions. Further large-scale epidemiological investigations, with standard sampling methods and adequate power, should be conducted to gain a more precise estimate of the status of the HIV and other sexually transmitted infection epidemics among older MSM in China.

## Electronic supplementary material

Additional file 1: Quality assessment checklist for observational studies (QATSO Score) concerning HIV prevalence/risk behaviours among MSM.(DOC 82 KB)

Below are the links to the authors’ original submitted files for images.

Authors’ original file for figure 1

Authors’ original file for figure 2

Authors’ original file for figure 3

Authors’ original file for figure 4

Authors’ original file for figure 5

## Abbreviations

HIV: Human immunodeficiency virus
AIDS: Acquired immune deficiency syndrome
UAI: Unprotected anal intercourse
MSM: Men who have sex with men
STI: Sexually transmitted infection.
