# Supplementary material for: High prevalence of HIV infection and unprotected anal intercourse among older men who have sex with men in China: a systematic review and meta-analysis
Source: BMC Infect Dis. 2014 Oct 6;14:531. doi: 10.1186/1471-2334-14-531 (PMC4287343; doi:10.1186/1471-2334-14-531)
Supplement: Supplementary file 1 — Additional file 1: Quality assessment checklist for observational studies (QATSO Score) concerning HIV prevalence/risk behaviours among MSM.(DOC 82 KB) [file 12879_2014_3858_MOESM1_ESM.doc]

**Table S2. Quality assessment checklist for observational studies (QATSO Score) concerning HIV prevalence/ risk behaviours among MSM**

| First author, published year | 1. Was the sampling method representative of the population intended to the study? | 2. Was the measurement of HIV objective (if the article is focusing only on risk behaviour among MSM, please select “Not applicable” for this question)? | 3. Did the study report any response rate? | 4. Did the investigator(s) control for confounding factors when analyzing the associations？ | 5. Was privacy or sensitivity of the nature of HIV considered when the survey was conducted? | QACO score  (0%-33%: Bad; 34%-66%: Satisfactory; 67%-100%: Good) |
| --- | --- | --- | --- | --- | --- | --- |
| Ma AB,  2007 | Non-probability sampling | By clinical records or lab tests | Yes | NA | Yes | 75%: Good |
| Xi SJ,  2011 | Non-probability sampling | By clinical records or lab tests | Yes | NA | Yes | 75%: Good |
| Long QP,  2012 | Non-probability sampling | By clinical records or lab tests | Yes | NA | Yes | 75%: Good |
| Zhou JB,  2012 | Non-probability sampling | By clinical records or lab tests | Yes | NA | Yes | 75%: Good |
| Wang ZC,  2012 | Non-probability sampling | By clinical records or lab tests | Yes | NA | Yes | 75%: Good |
| Qun He,  2006 | Non-probability sampling | By clinical records or lab tests | Yes | NA | Yes | 75%: Good |
| Xuan ZB,  2012 | Non-probability sampling | By clinical records or lab tests | Yes | NA | Yes | 75%: Good |
| Chu ZX,  2011 | Non-probability sampling | By clinical records or lab tests | Yes | NA | Yes | 75%: Good |
| Yan Xiao,  2010 | Non-probability sampling | By clinical records or lab tests | Yes | NA | Yes | 75%: Good |
| Wu ZY,  2013 | Non-probability sampling | By clinical records or lab tests | Yes | NA | Yes | 75%: Good |
| Zhou YQ,  2012 | Non-probability sampling | By clinical records or lab tests | Yes | NA | Yes | 75%: Good |
| Yang LG,  2012 | Non-probability sampling | By clinical records or lab tests | Yes | NA | Yes | 75%: Good |
| Lan GH,  2009 | Non-probability sampling | By clinical records or lab tests | Yes | NA | Yes | 75%: Good |
| Feng F,  2009 | Non-probability sampling | By clinical records or lab tests | Yes | NA | Yes | 75%: Good |
| Zhang FX,  2011 | Non-probability sampling | By clinical records or lab tests | Yes | NA | Yes | 75%: Good |
| Li R,  2010 | Non-probability sampling | By clinical records or lab tests | Yes | NA | Yes | 75%: Good |
| Chen Y,  2013 | Non-probability sampling | By clinical records or lab tests | Yes | NA | Yes | 75%: Good |
| Ni ZM,  2011 | Non-probability sampling | By clinical records or lab tests | Yes | NA | Yes | 75%: Good |
| Xu J,  2010 | Non-probability sampling | By clinical records or lab tests | Yes | NA | Yes | 75%: Good |
| Zheng LX,  2012 | Non-probability sampling | By clinical records or lab tests | Yes | NA | Yes | 75%: Good |

**Additional File 1. Quality assessment checklist for observational studies (QATSO Score) concerning HIV prevalence/ risk behaviours among MSM**

1. Was the sampling method representative of the population intended to the study?

| A. | Non-probability sampling (including: purposive, quota , convenience and snowball sampling) | 0 |
| --- | --- | --- |
| B. | Probability sampling (including: simple random, systematic, stratified g, cluster, two-stage and multi-stage sampling) | 1 |

1. Was the measurement of HIV objective (if the article is focusing only on risk behaviour among MSM, please select “Not applicable” for this question)?

| A. | By questionnaires (Self-reported) | 0 |
| --- | --- | --- |
| B. | By clinical records or lab tests | 1 |
| C. | Not applicable | NA |

1. Did the study report any response rate? (If the reported response rate is below 60%, the question should be answered “No”.)

| A. | No | 0 |
| --- | --- | --- |
| B. | Yes | 1 |

1. Did the investigator(s) control for confounding factors (e.g. stratification/ matching/ restriction/ adjustment) when analyzing the associations (if the study contains purely descriptive results, no association and prediction tests were conducted in the test, please select “Not applicable”)?

| A. | No | 0 |
| --- | --- | --- |
| B. | Yes | 1 |
| C. | Not applicable | NA |

1. Was privacy or sensitivity of the nature of HIV considered when the survey was conducted eg if conducted in a non-MSM or general clinic setting?

| A. | No | 0 |
| --- | --- | --- |
| B. | Yes | 1 |

Scoring method: Total score divided by total number of all applicable items

Grading of the QACO score:

| 0% -33% | 33%- 66% | 67% -100% |
| --- | --- | --- |
| Bad | Satisfactory | Good |
