# Supplementary figures and images for: High prevalence of HIV infection and unprotected anal intercourse among older men who have sex with men in China: a systematic review and meta-analysis
Source: BMC Infect Dis. 2014 Oct 6;14:531. doi: 10.1186/1471-2334-14-531 (PMC4287343; doi:10.1186/1471-2334-14-531)

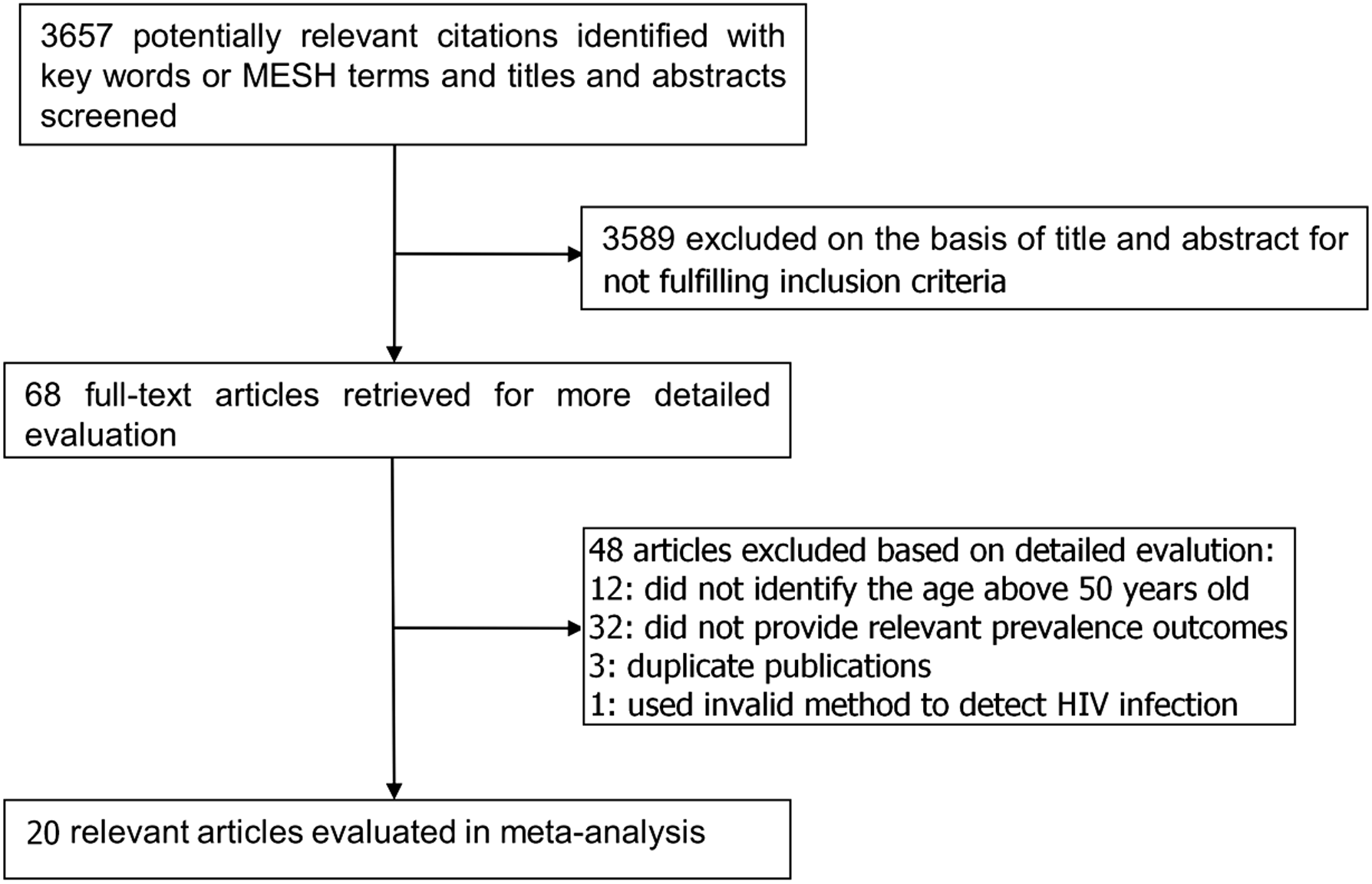

Supplement: Supplementary file 2 — Authors’ original file for figure 1 [file 12879_2014_3858_MOESM2_ESM.tif]

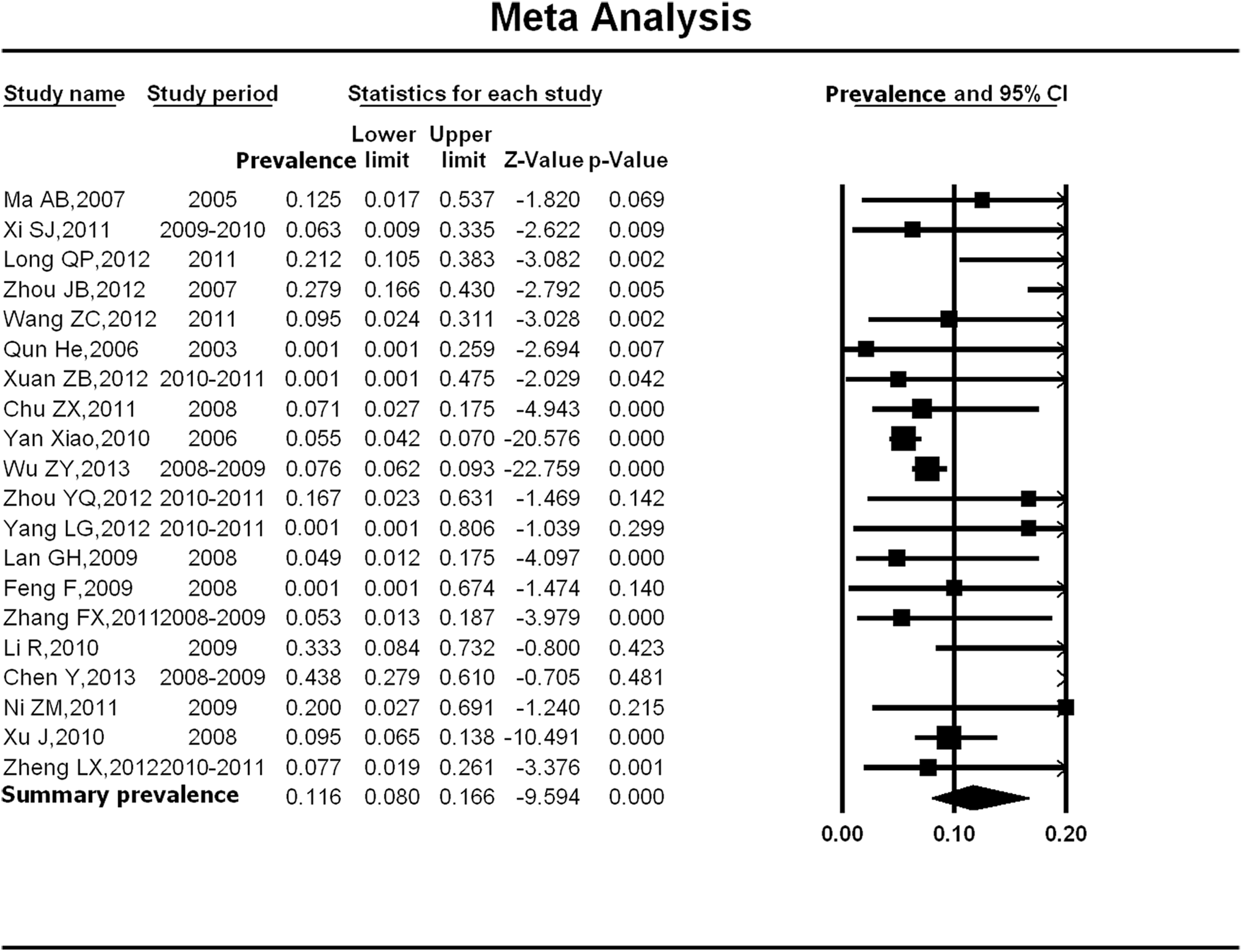

Supplement: Supplementary file 3 — Authors’ original file for figure 2 [file 12879_2014_3858_MOESM3_ESM.tif]

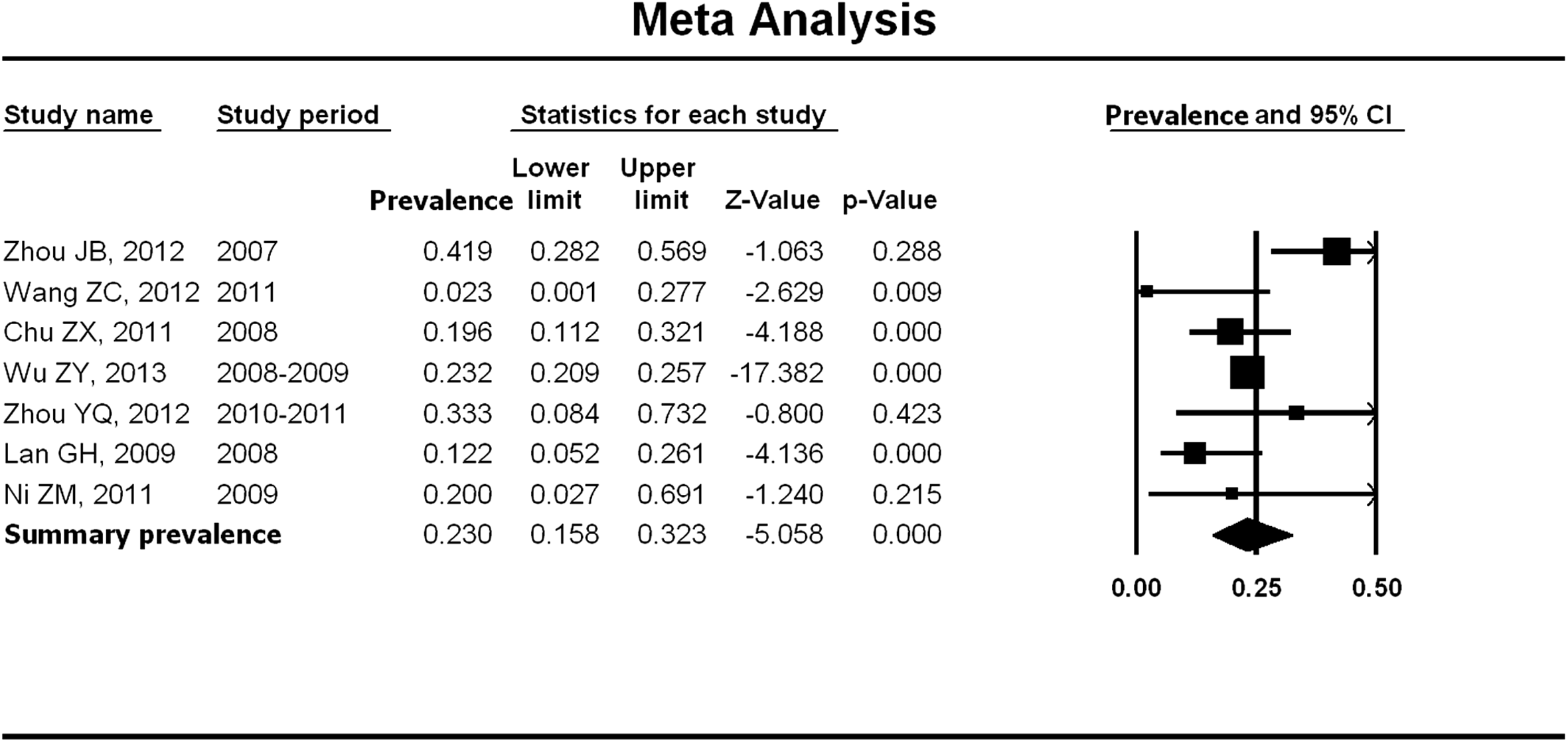

Supplement: Supplementary file 4 — Authors’ original file for figure 3 [file 12879_2014_3858_MOESM4_ESM.tif]

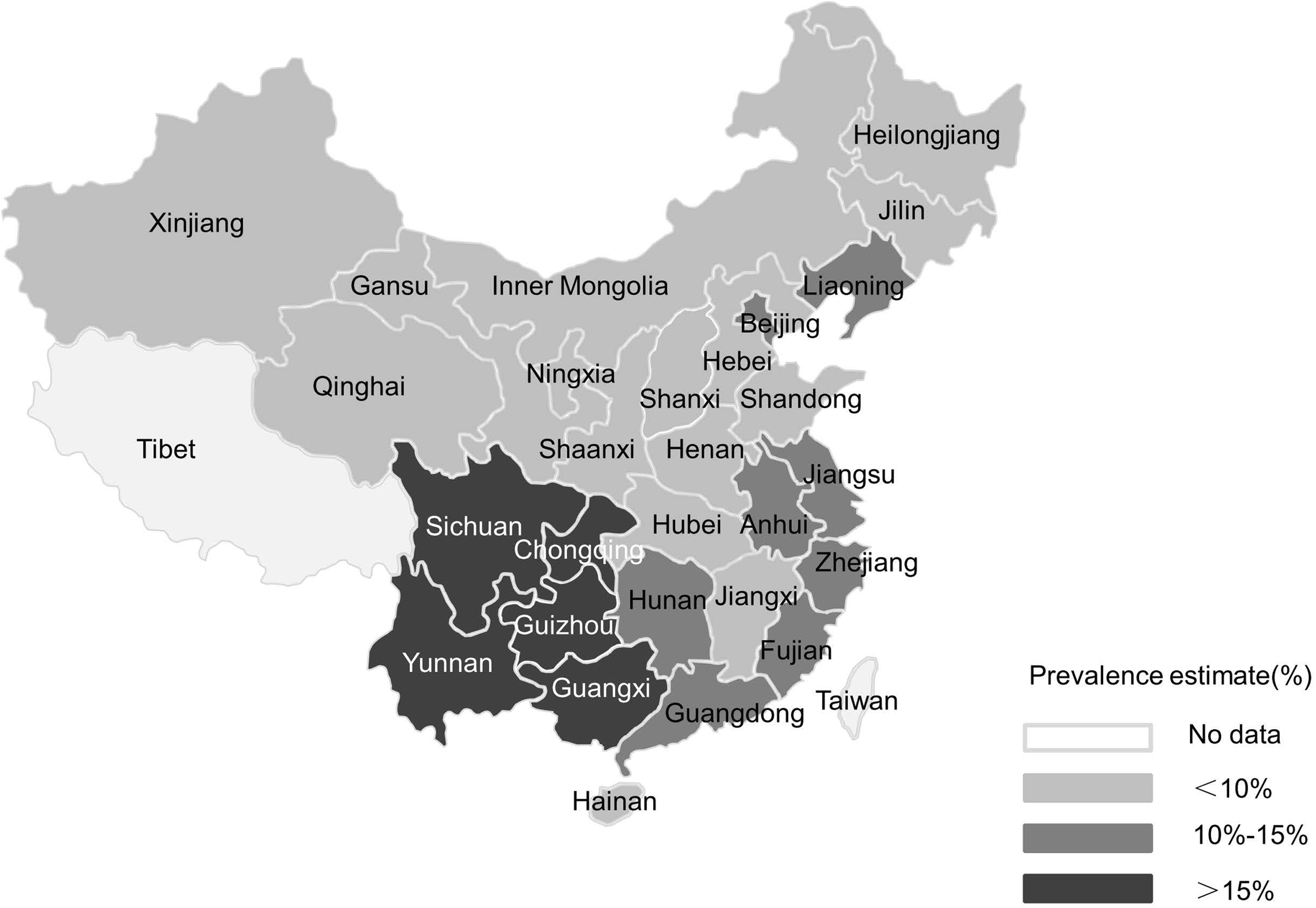

Supplement: Supplementary file 5 — Authors’ original file for figure 4 [file 12879_2014_3858_MOESM5_ESM.tif]

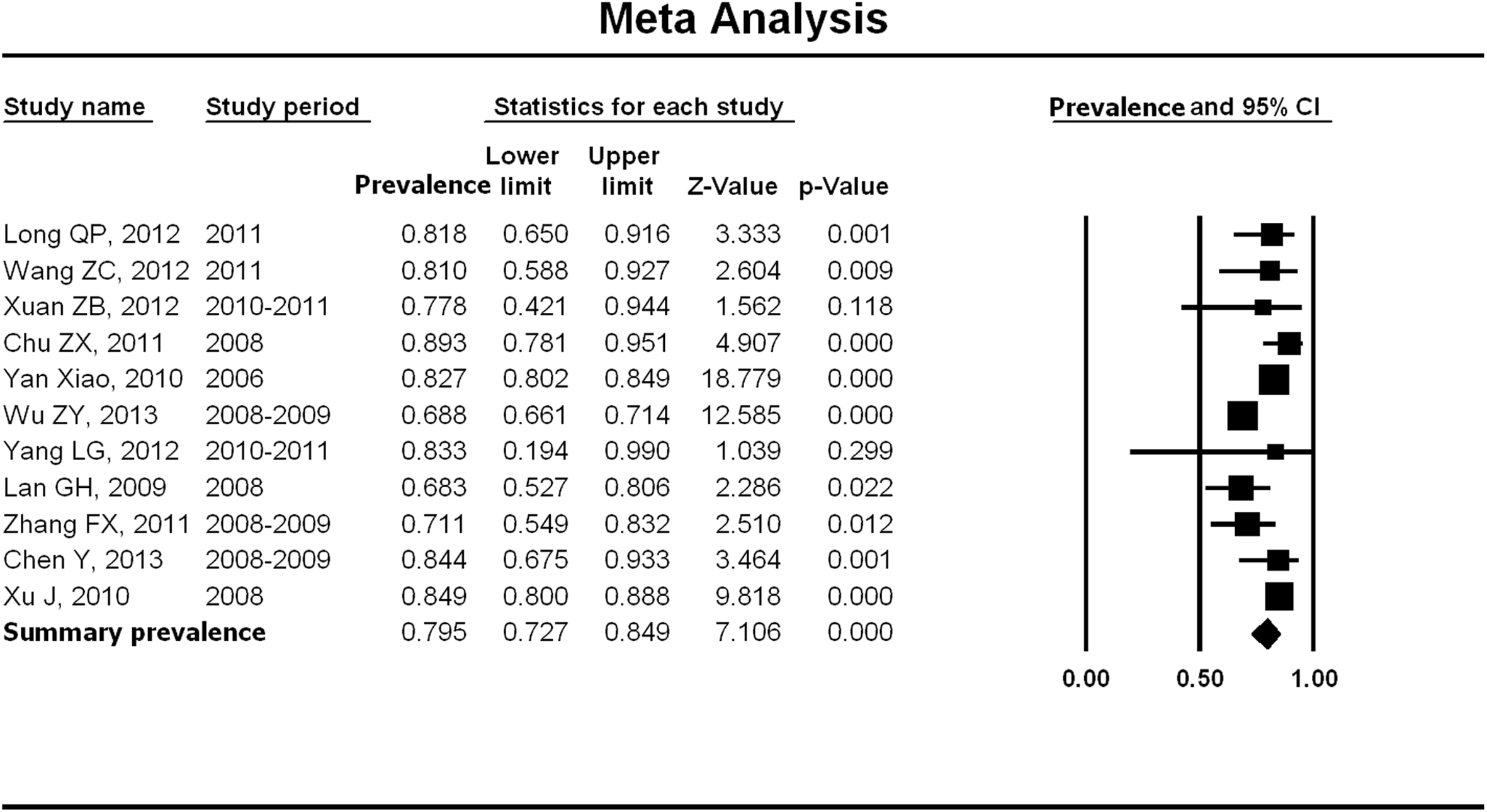

Supplement: Supplementary file 6 — Authors’ original file for figure 5 [file 12879_2014_3858_MOESM6_ESM.tif]
